# Characteristics of pain in amyotrophic lateral sclerosis

**DOI:** 10.1002/brb3.296

**Published:** 2015-01-21

**Authors:** Frank Hanisch, Anika Skudlarek, Janine Berndt, Malte E Kornhuber

**Affiliations:** Department of Neurology, Martin-Luther-University Halle-WittenbergErnst-Grube-Str. 40, Halle (Saale), D-06120, Germany

**Keywords:** Amyotrophic lateral sclerosis, cramps, motor neuron disease, pain, spasticity

## Abstract

**Background:**

Pain is an often underestimated and neglected symptom in amyotrophic lateral sclerosis (ALS).

**Methods:**

In a cross-sectional survey, 46 patients with ALS, 46 age- and gender matched population-based controls, and 23 diseased controls with myotonic dystrophy type 2 (DM2) were screened for occurrence, type, distribution, and treatment of pain and cramps. Data were collected with the use of the short form brief pain inventory (BPI).

**Results:**

Pain was reported in 78% of ALS patients,79% of DM2 patients, and 54% of controls (*P* < 0.05). More ALS patients than controls reported moderate to severe pain (42% vs. 20%). Pain in ALS patients interfered significantly more with daily activities than in controls (median pain interference score: 3.0 vs. 1.2, *P* < 0.05), especially enjoyment of life (5.0 vs. 1.0) and mood (3.0 vs. 1.0). There was no correlation between the duration of the disease and the severity of pain. Movement-induced cramps were reported in 63% of ALS patients, mostly in the distal extremities. There was no difference in the duration of ALS disease between patients reporting cramps and those who did not.

**Discussion:**

Our study showed that pain was a relatively frequent symptom which had an important impact on the quality of life. Pain that requires treatment can occur at every stage of ALS.

## Introduction

The progressive loss of upper and lower motor neurons in amyotrophic lateral sclerosis (ALS) causes a rapidly advancing paresis and atrophy of skeletal muscles which is occasionally combined with spasticity. Pain has been considered relatively rarely in ALS, in particular in the early stages of the disease. It has been suggested that the intensity of pain increases with disease duration and decreasing functional status (Drory et al. 2001; Chiò et al. 2012). Pain has been shown to be associated with a decreased quality of life and with a higher rate of depression. Recently, pain was reported by 51–72% of ALS patients in different populations in cross-sectional surveys (Chiò et al. 2012; Pagnini et al. 2012; Pizzimenti et al. 2013). Since pain in ALS was firstly reported it took about 30 years to draw the attention of physicians to this topic (Drake 1983).

Musculo-skeletal pain may arise as a result of stress on bone and joints that have lost their protective muscular sheath due to atrophy. In addition, pain was found to be associated with muscle contractures, reduced joint mobility, muscle cramps, spasticity, and skin pressure caused be immobility (Borasio and Voltz 1997; Brettschneider et al. 2013). Pain might also occur as a manifestation of a small-fiber neuropathy which was found in skin biopsies in 79% of ALS patients (Weis et al. 2011). Currently, pain is frequently underestimated and insufficiently treated.

The objective of this study was to determine prevalence, severity, interference, site, type of pain and its treatment in 46 German patients with ALS by using the brief pain inventory (BPI) and to correlate it with disease duration and severity parameters. We also analyzed the prevalence, site, and treatment of cramps and spasticity.

## Materials and Methods

### Patients

Patients were eligible if they fulfilled the criteria of probable and definitive ALS according to the revised El Escorial criteria (Brooks et al. 2000). Patients who had signs and symptoms of (frontotemporal) dementia were excluded (*n* = 4). All consecutive patients were seen by author F.H. at the Outpatient Clinic for ALS and Motoneuron Diseases, Department of Neurology, Martin-Luther-University, Halle (Germany) between March 2013 and September 2013 and were asked to fill out the questionnaires during a visit to the clinics for follow-up. All patients were on riluzole and received physiotherapy in regular intervals, for example, once weekly. The study was approved by the local ethics committee of the Martin-Luther University Halle. All participants provided written informed consent.

Population-based controls, who had to be free of ALS, were either partners or acquaintances of ALS patients, and patients with genetically confirmed myotonic dystrophy type 2 (disease-control group) were asked to fill out the short form of the BPI only.

### Pain assessment

The short form of the BPI is a self-administered 9-item questionnaire that was used to assess the presence and severity of current pain (pain within the previous 24 h) and its interference with daily activities. Pain was rated from 0 (no pain) to 10 (pain as bad as you can imagine). Four questions asked patients to rate the worst, least, and average pain experienced in the previous 24 h, and also to rate current pain. The average of these four answers was used to produce a pain severity score (PSS). The recall bias of the BPI is minimal. Finally, the BPI assesses the sites of pain and its treatment (Cleeland and Ryan 1994). The BPI has been shown to have good reliability and validity with patients with malignant and nonmalignant pain. It has been widely used to evaluate pain in neuromuscular disorders including ALS (Jensen et al. 2005; Chiò et al. 2012; Suokas et al. 2012; Güngör et al. 2013). The BPI questionnaire was available in German. In addition, patients were asked for the occurrence, frequency, and site of cramps and spasticity and its therapy.

### Functional assessment

The revised ALS functional rating scale (ALS-FRS-R; range: 0–48) was used to score activities of daily living. A lower score means lower functional abilities (Cedarbaum et al. 1999). Muscle strength was measured according to the Medical Research Council (MRC) scale (grades 0–5). The MRC sum score was calculated by summing the MRC grades of 18 muscles (maximum 170). Vital capacity to assess the pulmonary function was measured in sitting position (EasyOne™ diagnostic spirometer; nnd Medizintechnik AG, Zürich, Switzerland).

### Statistics

Descriptive statistics were used to summarize all variables for the patient and control groups. To assess differences in demographic characteristics and differences in the prevalence, severity, interference, and treatment of pain between patients and population-based controls, we used the Chi-squared trend test for discrete data, or the Mann–Whitney *U* test for continuous data, and the two-tailed Fisher-Exact-test. Differences between the three groups were analyzed using Kruskal–Wallis One Way Analysis of Variance on Ranks, post hoc analysis was carried out using all pairwise multiple comparison procedures (Holm–Sidak method or Dunn's method). A significance level of 0.05 was used. All analyses were performed using SPSS for Windows (version 2.0; SPSS Inc., Chicago, IL).

## Results

### Demographic data

We invited 55 ALS patients to participate in the survey, 46 of whom took part. The response rate was 84%. The demographic and clinical profiles are listed in Table 1. ALS patients had a median age of 64.0 years (range 31–79), the median disease duration since onset of paresis was 19 months (range: 3–102). Fifty-six percent of ALS patients were female. There were no significant differences between age of ALS and DM2 patients and controls. Fifteen of 46 ALS patients received occupational therapy.

**Table 1:** Questionnaire-based demographic and clinical data of 46 patients with amyotrophic lateral sclerosis (ALS). Data are given either as percentage or median (range)

Parameter	ALS patients
Female, *n* (%)	26 (57)
Median onset of ALS (years)^1^	62.0 (29–77)
Bulbar/Extremity onset	10/35
Probable/definite ALS	29/17
Median duration of ALS at examination, range (months)	19.0 (3–102)
Median ALS-FRS-R Score, range	33.1 (5–45)
Assessment of treatment for pain	
Patients receiving treatment for pain, *n* (%)	17 (37)
NSAID, *n* (%)	8 (17)
Opiates/Opioides, *n* (%)	6 (13)
Tricyclic antidepressants, *n* (%)	0 (0)
Katadolone, *n* (%)	1 (2)
Antiepileptic drugs, *n* (%)	3 (6)
Specific physiotherapy, *n* (%)	2 (4)
Assessment of treatment for crampi	
Crampi, reported, *n* (%)	29 (63)
Patients receiving treatment for crampi, *n* (%)	12 (26)
Treatment with	
Magnesium (%)	12 (26)
Chinine sulfate (%)	2 (4)
Pregabaline *n* (%)	1 (2)
Assessment of treatment for spasticity	
Spasticity, in neurological examination, *n* (%)	5 (11)
Patients receiving treatment for spasticity, *n* (%)	
With baclofen, *n* (%)	2 (4)
With tolperisone, *n* (%)	1 (2)

ALS-FRS-R revised version of the amyotrophic lateral sclerosis functional rating scale, MRC Medical Research Council, NSAID nonsteroidal anti-inflammatory drugs.

1 Defined as onset of paresis reported by the patient.

A history of diabetes had 5/46 ALS patients, 11/23 DM2 patients, and 4/46 population-based controls.

### Prevalence, severity, and interference of pain

Seventy-eight percent of the 46 patients (*n* = 36) reported having pain, against 54% of controls (*n* = 25); *P* = 0.027). These figures obtained with the BPI short-form refer to the prevalence of pain in the last 24 h.

Table 2 shows the severity of pain and its interference with daily life in patients and controls. On a scale from 0 to 10, the median pain severity score (PSS) among patients reporting pain in the previous 24 h was 3.0 (range 0.5–6.8). For controls it was 2.0 (range 0.5–5.3), and did not significantly differ from patients (*P* = 0.08).

**Table 2:** Characteristics of pain (PSS; pain related interference with daily activities) as reported in patients with ALS and age- and gender-matched population-based controls. Data are given either as percentages or median (range). *Differences between groups were analyzed using Kruskal–Wallis One Way Analysis of Variance on Ranks, post hoc analysis was carried out using all pairwise multiple comparison procedures (Holm–Sidak method)

	ALS	DM2	Controls	*P*
Men/women (%)	20/26	19/4	20/26	
Mean age (years)	64.0±10.8 (31–79)	60±10.7 (27–77)	60.0±10.1 (39–77)	n.s.*,^a^
Reporting pain (Number (%) out of total population)	36 (78)	19 (83)	25 (54)	
Pain severity (0–10)				
Median PSS (range)	3.0 (0.5–6.8)	2.8 (0–6.0)	2.0 (0.5–5.3)	n.s.*,^a^
PSS subgroups				
No pain (rating of 0) [*n* (%)]	–	–	–	
Mild pain (1–3) [*n* (%)]	21 (58)	9 (47)	20 (80)	
Moderate pain (4–6) [*n* (%)]	14 (39)	10 (53)	5 (20)	
Severe pain (7–10) [*n* (%)]	1 (3)	0 (0)	0 (0)	
Pain related interference with daily activities (0–10)				
Median pain interference score (range)	3.0 (0.75–6.75)	3.4 (0–6.6)	1.2 (0.75–5.25)	<0.05*,^b^
General activity, median (range)	3.0 (0–10)	4.2 (0–10)	2.0 (0–5)	<0.05*,^c^
Mood, median (range)	4.0 (0–10)	3.1 (0–10)	1.0 (0–5)	<0.01*,^b^
Walking ability, median (range)	3.0 (0–10)	4.2 (0–9)	1.0 (0–7)	<0.05*,^b^
Normal work, median (range)	2.5 (0–10)	4.7 (0–10)	1.0 (0–8)	<0.05*,^b^
Relations with other people, median (range)	1.5 (0–10)	1.6 (0–5)	0 (0–3)	<0.05*,^b^
Sleep, median (range)	3.0 (0–9)	3.6 (0–10)	2.0 (0–9)	n.s.*,^a^
Enjoyment of life, median (range)	5.0 (0–10)	2.9 (0–9)	1.0 (0–6)	<0.01*,^b^
Treatment for pain *n* (%)	17 (47)	9 (47)	11 (43)	

ALS amyotrophic lateral sclerosis, DM2 myotonic dystrophy type 2, PSS pain severity score.

* Post hoc analysis using all pairwise multiple comparison procedures (Holm–Sidak method or Dunn's method):

a no difference between groups,

b *P* < 0.05 ALS and DM2 versus controls,

c *P* < 0.05 DM2 versus controls.

At 3.0 (range 0.75–6.75), ALS patients' median Pain Interference Score (PIS) differed significantly from that of controls (PIS 1.2 (range 0.75–5.25); *P* = 0.01). Pain interfered especially with patients' enjoyment of life (score of 5) and mood (score of 4), followed by mild interference with general activities, walking ability, and sleep (score of 3). Relationships with other people were the least affected (score of 1.5). For each of the seven domains of daily life, the interference scores were significantly worse in ALS patients than in controls, except for interference with sleep and the general activity.

### Sites and type of pain, and treatment

**Figure 1:**
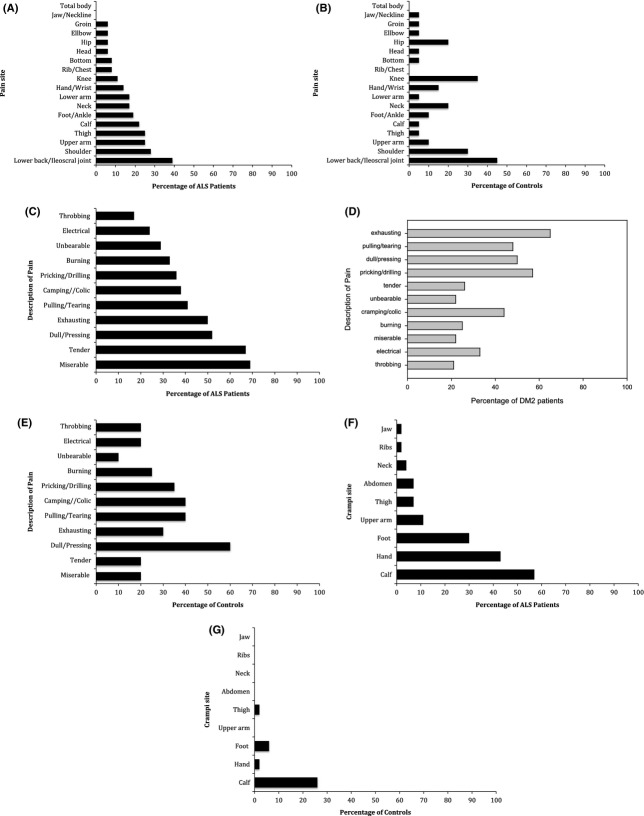
Sites of pain (in/across different parts of the body) in 36 patients with amyotrophic lateral sclerosis (ALS) (A) and in 25 controls (B) reporting pain in the last 24 h. Description of pain in 36 patients with ALS (C), 19 patients with DM2 (D), and 25 controls (E) reporting pain in the last 24 h. Sites of crampi (in/across different parts of the body) in 46 patients with ALS (F) and in 25 controls (G). Data for patients with DM2 were not available.

Figure1A and B shows the reported sites of pain in ALS patients and controls reporting pain in the last 24 h. Fifty-three percent of the ALS patients with pain reported pain in more than one site. The back (50%), the extremities (47%), and the joints (42%) were most affected. In DM2 patients pain was mostly located proximally (i.e., shoulder and hip girdle, back and upper leg; data not shown).

Controls reported pain frequently in the back (45%) and in joints (50%), but rarely in the extremities (10% of controls). There was no significant difference in the painful affection of neck, shoulder, elbow, hip, knee, and ankles between ALS patients, DM2 patients, and controls.

Figure1C–E depicts the way in which pain was described by those patients and controls who reported pain in the last 24 h. The commonest words ALS patients used to describe pain was “miserable” (69%, controls: 20%), “tender” (67%, controls: 20%), “dull/pressing” (52%, controls: 60%), and “exhausting”(50%, controls: 30%). In DM2 the qualities “exhausting” (65%), “pricking/drilling” (57%), and “dull/pressing” (50%) were most commonly mentioned.

Forty-seven percent of ALS patients with pain (*n* = 17) reported receiving treatment for it (Table 1). Seventeen used medication only (mainly nonsteroidal anti-inflammatory drugs (NSAIDS), but also opiates, antiepileptic drugs, and others). In addition, two patients received acupuncture, massage, and ultrasound to relieve pain. Nineteen percent of the ALS patients with mild pain used some kind of pain therapy, against 87% of the ALS patients with moderate to severe pain. Forty percent of ALS patients with moderate to severe pain took opiates. Compared to ALS patients, a similar percentage of controls reporting pain used treatment for it (43%, *n* = 11). Four controls used medication only, four used physical therapy only, and three took a combination of both.

### Association of pain with patients' demographic and clinical characteristics

There was no correlation between the duration of the disease and the severity of pain (PSS; *r* = 0.15). Those patients with none or mild pain in the PSS (score 0–3) had a similar disease duration compared to patients with moderate or severe pain in the PSS (score 4–10) (20 weeks vs. 18.5 weeks, n.s.).

There was a weak negative correlation *r* = −0.47 between the ALS-FRS-R and the pain severity scale (PSS; *P* < 0.05).

### Prevalence and sites of cramps, and treatment

Sixty-three percent of the 46 ALS patients reported having cramps (Table 1). Thirty-three percent received treatment for it, mainly magnesium (26%). Figure1F shows the reported sites of cramps in ALS patients. The calf (57%), the hands and fingers (43%), and the feet and toes (30%) were most affected by cramps. The cramps were mainly movement-induced, frequently short-lasting, and reported to occur both during daytime and at night.

Twenty-eight percent of the controls reported having cramps occasionally (Fig.1G). Cramps occurred mostly in the calf (26%), rarely in the foot (6%). The cramps were reported to occur most frequently during the night and were less frequently provoked by movement during daytime. Patients with DM2 were not asked for the occurrence of cramps.

### Association of cramps with patients' demographic and clinical characteristics

There was no difference in the duration of ALS disease between (A) patients reporting cramps and those who did not, and (B) patients reporting to take drugs for cramps and those who did not (data not shown).

The ALS-FRS-R in patients reporting cramps (score 38.5) was nonsignificantly higher compared to those reporting no cramps (score 34.5; *P* = 0.4). Fifteen of 31 patient (49%) with none or mild pain in the PSS (score 0–3), and 12 of 15 patients (80%) with moderate to severe pain (PSS score 4–10) reported to experience cramps. Ten out of 15 patients reporting none or mild pain took medication for cramps and 2 out of 12 patients reporting moderate to severe pain (Table 1).

### Prevalence of spasticity and treatment

On neurological examination, spasticity was present in 11% of the 46 patients with ALS (Table 1, legs *n* = 5 arms *n* = 1). Six percent required treatment either with baclofen or tolperisone.

## Discussion

### Pain

In this cross-sectional study 78% of ALS patients with a median disease duration of 19 months and a median ALS-FRS-R of 33 reported some degree of pain during the last 24 h. This is slightly higher compared to previous studies reporting pain in 37–72% of ALS patients with a disease duration of 15–31 months and ALS-FRS-R between 28 and 35 points (Chiò et al. 2012; Pagnini et al. 2012; Pizzimenti et al. 2013; Rivera et al. 2013). This is considerably higher than the occurrence of pain as reported by patients with Pompe disease (45%; Güngör et al. 2013) and similar to the according percentage in patients with myotonic dystrophy type 2 (79%) using the BPI. Thirty-three percent of all ALS patients reported moderate to severe pain in this study. This is in line with previous reports reporting moderate to severe pain in 14–36% of ALS patients (Chiò et al. 2012; Pagnini et al. 2012; Wicks 2012). The 31% of ALS patients reporting pain in our retrospective chart analysis rather reflects moderate and severe pain only. Without using a sensitive questionnaire, mild pain intensities would easily be missed in an interview. This may be the reason for the wide range of the presence of pain in ALS patients as reported by previous investigations (Drory et al. 2001; Chiò et al. 2012; Pagnini et al. 2012; Wicks 2012; Pizzimenti et al. 2013; Rivera et al. 2013).

It was suggested that muscle atrophy may cause stress on bone and joints that have lost their protective muscular sheath, hence causing pain. But in this study, we could not find a higher frequency of pain located in joints (neck, shoulder, elbow, wrist, hip, knee, and ankle) in ALS patients as compared to controls. However, consistent with Chiò et al. (2012), pain was statistically more often located in the extremities (both arms and legs) by ALS patients compared to controls. This could be attributed to physical overexertion following progressing paresis and atrophy but also to the effect of longer lasting or severe cramps in mainly distal and small muscles. Fasciculations and crampi can result of an increased excitability of motor units due to axonal sprouting.

Among patients with different neuromuscular disorders, patients with ALS reported the greatest pain interference in the BPI (Jensen et al. 2005). In this study, the greatest interference of pain was found with mood and the enjoyment of life. This finding confirms the results of Chiò et al. (2012), who also used the BPI. This is in line with the finding that pain in ALS is associated with depressive symptoms which are significantly related to a poorer quality of life (Tedman et al. 1997; Pizzimenti et al. 2013). The influence of pain on the quality of life and on the presence of depressive symptoms in ALS patients stresses the eminent importance to inquire signs and symptoms of pain during follow-up. Pain was seldom mentioned as the worst aspect of the disease using a standardized interview compared to “loss of speech”, “loss of mobility”, and “the poor prognosis” (Hecht et al. 2002). This study using the BPI cannot answer the question whether pain is among the worst or most disabling symptoms (1) at the beginning or later stages of the disease or (2) whether pain is important in some patients but replaced in the awareness of both physician and patient by equally or even more disabling symptoms dominating in the later course of the disease (e.g., loss of speech and swallowing, respiratory problems, and loss of ambulance). It should be kept in mind that pain was found to be the most significant contributor to suffering in the final stages of disease (Ganzini et al. 1999).

Previously, pain frequency and intensity were correlated with a worse functional score and with a longer disease duration (Hecht et al. 2002; Chiò et al. 2012). While the negative correlation between pain intensity and functional status in the ALS-FRS-R was reproduced, no difference in the presence of pain among patients in the different stages of ALS was found, that is, pain was also present in the early stages of disease (Rivera et al. 2013).

The most frequent qualities of pain mentioned by ALS patients in this study were “miserable”, “tender”, “dull/pressing”, and “exhausting”, whereas the most frequently mentioned pain qualities in Pompe disease, myotonic dystrophy type 2, and normal controls were “exhausting”, “pulling/tearing”, “dull/pressing”, and “pricking/drilling” (Güngör et al. 2013; F. Hanisch unpubl. results). The description of pain does not seem to be sufficient to discriminate ALS patients from patients with myopathies or normal controls (Pagnini et al. 2012). It could be hypothesized that “tender” reflects a neuropathic quality. Previous studies using other questionnaires mentioned the qualities “dull” and “electrical” (Newrick and Langton-Hewer 1985) and “nagging”, “sore”, “periodic” (Pagnini et al. 2012). A small-fiber neuropathy in some patients with ALS might contribute or cause this types of pain (Weis et al. 2011). However, if pain in our ALS patients were related to small-fiber neuropathy, the distribution of pain would have been expected to be distal in the limbs.

In this study 37% of all ALS patients (i.e., 47% of patients reporting pain) required specific pain treatment, it is noteworthy that 13% were treated with opioids or opiates. In other case series the percentage of patients receiving therapy for pain was even higher (Chiò et al. 2012).

A recent Cochrane review draw the disillusioned conclusion that no randomized or quasirandomized controlled trials on drug therapy for pain in ALS or motor neuron disease is currently available (Brettschneider et al. 2013). It was recommended to follow the treatment guidelines of the WHO Analgetic Ladder (WHO 1990). Cramps and spasticity but not pain was mentioned in the EFNS guidelines on the Clinical Management of ALS in 2012 (EFNS Task Force on Diagnosis and Management of Amyotrophic Lateral Sclerosis 2012).

### Cramps

At the time of the survey 63% of ALS patients were reporting cramps. In contrast to normal controls, in which cramps mostly occur in the calves and during night, cramps in ALS patients are frequently located in the distal small muscles of toes/feet and in those of fingers/hands, are short-lasting and movement-induced and therefore interfere with functional activities. The occurrence of cramps in this study was not associated with the duration of the disease, suggesting that cramps are a permanent symptom as long as some functional abilities are preserved.

In this study only half of the patients who reported pain required some drug treatment for it. In the majority, magnesium was sufficient to control the cramps and only four patients were treated with either quinine sulfate or with antiepileptic drugs. There is a paucity of data about the frequency and severity of cramps in ALS patients, their interference with the functional status and their development over time. This symptom is usually not considered in studies about pain in ALS (Drory et al. 2001; Chiò et al. 2012; Pagnini et al. 2012; Wicks 2012; Pizzimenti et al. 2013; Rivera et al. 2013). Compared to the analysis of pain, the evaluation of crampi is hampered by the lack of a standardized and validated instrument, for example, no questionnaire to address this symptom has been available yet.

A survey of the members of the European ALS Research Group had shown previously that for the treatment of cramps, quinine sulfate was used in 58% of centers, followed by benzodiazepines in 40%, magnesium in 25%, and carbamazepine in 23% (Borasio et al. 2001). Currently, possible effects of magnesium on exercise-associated muscle cramps or disease-state-associated muscle cramps have not been evaluated in randomized controlled trials (e.g., in patients with amyotrophic lateral sclerosis/motor neuron disease; Garrison et al. 2012). A Cochrane review found no consistent evidence for a benefit of magnesium in four placebo-controlled trials in patients with so-called idiopathic cramps, comprising mostly elderly patients with nocturnal cramps (Garrison et al. 2012). No favorable effect for any oral treatment of cramps in ALS/MND could be demonstrated in another Cochrane review analyzing randomized and quasi-randomized trials, but many studies were underpowered to draw a definite conclusion (Baldinger et al. 2012). An open label study described a favorable effect of gabapentin. So far, a randomized controlled trial with tetrahydrocannabiol was the only one choosing cramps as the primary endpoint in patients with ALS and moderate to severe cramps, but failed to show efficacy (Weber et al. 2010). According to the EFNS guidelines on the management of ALS, levetiracetam (1500 mg daily) as a first choice and quinine sulfate (200 mg twice daily) as a second choice alone or combined with physiotherapy and/or hydrotherapy may be tried (Bedlack et al. 2009; EFNS Task Force on Diagnosis and Management of Amyotrophic Lateral Sclerosis 2012).

### Limitations of the study

Limitations of our study comprise the relatively low number of patients, the cross-sectional design (instead of a long-term follow-up), the absence of a formal evaluation of depression and quality of life, and the lack of a standardized and validated questionnaire to assess cramps in ALS patients.

### Conclusion

Our study showed that pain was a relatively frequent symptom which had an important impact on the quality of life. Pain that requires treatment can occur at every stage of ALS. Treatment for pain should be recognized as an important aspect of palliative care in ALS. In the absence of any evidence from randomized controlled trials, the treatment of pain should follow the recommendations of the WHO Analgetic Ladder (WHO 1990). However, different causes of pain might require an individual approach (e.g., with antidepressants or antiepileptic drugs). There is a need for standardized and validated questionnaires to assess frequency and intensity of crampi and their response to therapies. In future, multicenter studies are warranted to analyze the effects of an efficient pain therapy on quality of life, depression, quality of sleep, and respiratory function.
